# Exon 3 mutations of *CTNNB1* drive tumorigenesis: a review

**DOI:** 10.18632/oncotarget.23695

**Published:** 2017-11-24

**Authors:** Chao Gao, Yingmei Wang, Russell Broaddus, Longhao Sun, Fengxia Xue, Wei Zhang

**Affiliations:** ^1^ Department of Gynecology and Obstetrics, Tianjin Medical University General Hospital, Tianjin, People's Republic of China; ^2^ Department of Pathology, The University of Texas MD Anderson Cancer Center, Houston, TX, USA; ^3^ Department of Cancer Biology, Wake Forest Baptist Comprehensive Cancer Center, Winston-Salem, NC, USA

**Keywords:** β-catenin, CTNNB1, mutations, Wnt/β-catenin signaling pathway, immunosuppression

## Abstract

The canonical Wnt/β-catenin signaling pathway, an important modulator of progenitor cell proliferation and differentiation, is highly regulated for the maintenance of critical biological homeostasis. Decades of studies in cancer genetics and genomics have demonstrated that multiple genes encoding key proteins in this signaling pathway serve as targets for recurrent mutational alterations. Among these proteins, β-catenin and adenomatosis polyposis coli (APC) are two key nodes. β-catenin contributes in transporting extracellular signals for nuclear programming. Mutations of the *CTNNB1* gene that encodes β-catenin occur in a wide spectrum of cancers. These mutations alter the spatial characteristics of the β-catenin protein, leading to drastic reprogramming of the nuclear transcriptional network. Among the outcomes of this reprogramming are increased cell proliferation, enhanced immunosuppression, and disruption of metabolic regulation. Herein we review the current understanding of *CTNNB1* mutations, their roles in tumorigenesis and discuss their possible therapeutic implications for cancer.

## INTRODUCTION

Cancer is a systems disease with a complicated pathogenesis. Tumorigenesis is characterized by abnormal regulation of cell growth and cell death. Mutations in proto-oncogenes and tumor suppressor genes underlie tumorigenesis by dysregulation of intracellular signaling pathways that are critical for the normal physiology of organisms [1]. The canonical Wnt/β-catenin signaling pathway is one such prominent pathway, being tightly regulated for maintenance of biological homeostasis. Frequently, this pathway is aberrantly activated in numerous cancers, including gastrointestinal, prostate, breast, and ovarian cancer [2–5]. Over the past several decades, studies of cancer genetics and genomics have demonstrated that multiple genes encoding key proteins in this signaling pathway are targets for recurrent mutational alterations. Among these proteins, β-catenin and Adenomatous polyposis coli (APC) are two key nodes that physically combine with each other in a complex. The tumor suppressor gene, *APC,* is one frequently mutated gene of Wnt signaling in human cancers [6]. As one of the central nodes, β-catenin contributes in transporting extracellular signals for nuclear programming [7]. β-catenin mutations may lead to constitutive activation of the Wnt/β-catenin signaling pathway and reprogramming of downstream nuclear transcriptional networks [8, 9]. In this review, we provide a focused overview of the integrated Wnt/β-catenin signaling pathway and the basic structure and biological roles of β-catenin. We also summarize the current understanding of β-catenin mutations in tumorigenesis and discuss their possible therapeutic implications for cancer.

## OVERVIEW OF THE CANONICAL WNT/β-CATENIN SIGNALING PATHWAY

The *Wnt* gene was first discovered in 1982 by Nusse and Varmus while studying the transcription mechanisms for a tumor virus in a murine mammary tumor [10]. Initially identified as *Int1*, the gene was determined to encode proteins that transfer growth and development signals between cells. Further studies demonstrated that this gene could make Drosophila flies wingless in normal embryonic development, and it was renamed *Wnt* [11, 12]. The Wnt protein family is cysteine-rich secreted glycoprotein with both autocrine and paracrine functions [13]. There are currently 19 identified members, including wnt1, wnt3A, and wnt5A [14–16]. Wnt signaling has proven to contribute on the embryonic development. The three highly characterized Wnt signaling pathways are the noncanonical Wnt-Ca2^+^ pathway, noncanonical planar cell polarity pathway, and canonical Wnt/β-catenin signaling pathway [13, 17–19]. In general, these pathways can be placed in two categories according to the presence or absence of β-catenin: canonical or noncanonical, respectively. Several other signal transduction pathways involve Wnt, such as the Wnt/Rac, Wnt/cAMP, and Wnt/Rho pathways [18, 20, 21].

**Figure 1:**
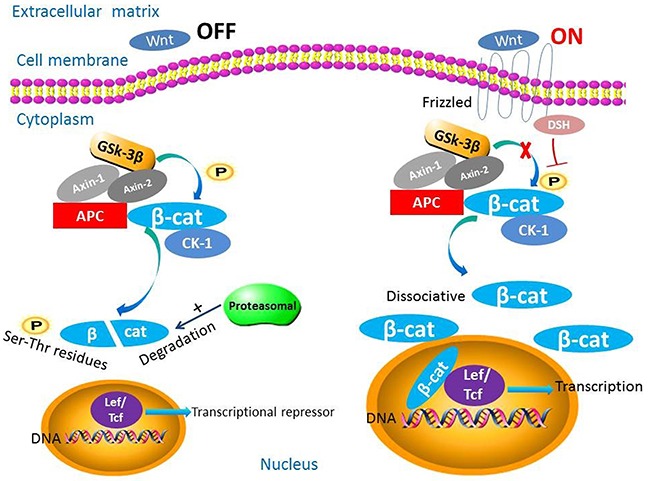
The biological roles of β-catenin in the Wnt/β-catenin signaling pathway This pathway has two states dependent upon the presence or absence of Wnt ligands. When Wnt ligands are absent, β-catenin is phosphorylated by the destruction complex and degraded. When Wnt ligands are present, β-catenin is not degraded and translocates to the nucleus and functions as a transcription factor.

The complexity of the canonical Wnt/β-catenin pathway derives from the high number of ligands and receptors involved in signaling that can elicit a variety of intracellular responses [22, 23]. As the key intracellular transducer of this pathway, β-catenin plays important roles in the entire process (Figure 1). Activity of β-catenin is controlled by the destruction complex, consisting of APC, AXIN-1, AXIN-2, casein kinase-1α (CK-1), protein phosphatase 2A (PP2A), and glycogen synthase kinase (GSK)-3β [7, 24–26]. This pathway has two states dependent upon the presence or absence of Wnt ligands.

In the absence of Wnt ligands, cytoplasmic β-catenin is phosphorylated at N-terminal serine-threonine residues by the destruction complex and degraded by the proteasome via the ubiquitin-proteasome pathway [7, 27]. In general, the ubiquitin-proteasome pathway involves three parts: a ubiquitin-activating enzyme, a ubiquitin-conjugating enzyme, and a ubiquitin ligase [28]. Without nuclear accumulation of β-catenin, nuclear T-cell factor/lymphoid enhancer factor (TCF/LEF) transcription factors associate with co-repressor proteins via their high-mobility group domains and act as transcriptional repressors. Authors reported that the co-repressor proteins contain Groucho/transducin-like enhancer of split and CREB-binding protein (CBP) [29–31].

Alternatively, in the presence of Wnt, ligand binds to the cell-surface receptor Frizzled and acts on Dishevelled protein [32, 33]. Frizzled is a seven-pass transmembrane protein with a long amino terminal extension called a cysteine-rich domain. The cysteine-rich domain is a special structure where Wnt proteins bind directly [15, 16, 34]. In addition to Frizzled, a long single-pass transmembrane molecule named low-density lipoprotein receptor-related protein (LRP) is bound to Wnt ligands. The identity of this protein is LRP5 or LRP6 in vertebrates and in Drosophila, a similar protein is derived from the arrow gene [35]. The cytoplasmic tail of LRP may combine with Axin directly [36, 37]. Other single-pass transmembrane proteins, such as receptor-like tyrosine kinase and receptor tyrosine kinase-like orphan receptor-1/2, can function as co-receptors, influencing Wnt signaling [38–40]. Moreover, numerous studies have suggested that the R-Spondin proteins also play potential roles in Wnt signaling and could stabilize the levels of cytosolic β-catenin and dramatically synergize with wnt3A [41–43]. Following Wnt binding, the protein kinases (A, B, and C), phosphoinositide 3-kinase (PI3K)/Akt, and mitogen-activated protein kinase (MAPK) inhibit the phosphorylation of GSK-3β. The destruction complex then becomes inactive, preventing β-catenin phosphorylation and destruction. Hypophosphorylated β-catenin accumulates in the cytoplasm and eventually translocates to the nucleus to function as a transcriptional factor, despite lacking a nuclear localization signal [44–47]. Thus, the networks of a broad spectrum of Wnt downstream target genes are programmed [7]. These target genes, such as cyclin D1, insulin-like growth factor-1, and CD44, are critical to some hallmarks of cancer such as epithelial-to-mesenchymal transition and metastasis [48, 49].

## OVERVIEW OF β-CATENIN AND ITS GENE MUTATIONS IN TUMORIGENESIS

### The primary and three-dimensional structures of β-catenin

**Figure 2:**
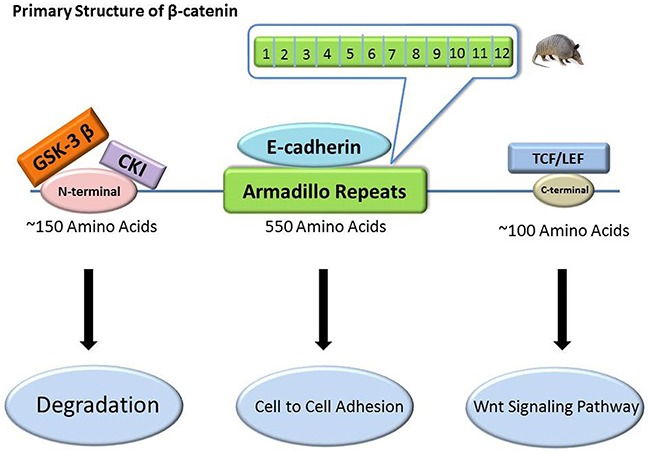
The primary structure of β-catenin and its relevant binding sites β-catenin has three domains: a 550-amino-acid central repeat domain, an approximately 150-amino-acid N-terminal domain, and an approximately 100-amino-acid C-terminal domain. They are binding sites for E-cadherin, GSK-3β/CK-1, and TCF/LEF, respectively, and exert different roles.

β-catenin is a multifunctional cytoplasmic protein composed of a polypeptide chain [50]. It is encoded by the gene *CTNNB1*, which maps to 3p21 [27] and with a size of 23.2 kb. *CTNNB1* has 16 exons according to restricted mapping and partial sequence analysis [51]. The primary structure of β-catenin consists of three domains: a 550-amino-acid central repeat, an approximately 150-amino-acid N-terminal domain, and an approximately 100-amino-acid C-terminal domain on both sides (Figure 2) [52–56]. The central repeat domain is also known as armadillo repeats [57]. The armadillo repeat domain consists of 12 armadillo repeats [58], each of which contains approximately 42 residues that form three helices arranged in a triangular shape. A superhelix, formed by these 12 contiguous repeats, features a long, positively charged groove. The third helix of each repeat constitutes the floor of this groove [58]. Generally, the N-terminal domain is the phosphorylation site for GSK-3β and casein kinase-1 as well as the binding site for α-catenin with the C-terminal domain involved in combination with nuclear (TCF/LEF) [59, 60]. The N- and C-terminal domains may combine with the armadillo repeat domain via a fold-back mechanism that may regulate the partner-binding properties of the armadillo repeat [61–64].

### The basic biological function of β-catenin

β-catenin is crucial for two important developmental processes: 1) establishment and maintenance of cell-type-specific cell-to-cell adhesion and 2) regulation of target gene expression via the Wnt signaling pathway [52, 65]. It also regulates the stem cell transcriptome via the Wnt, Notch, Hippo, and Hedgehog pathways [66–69]. In most cases, β-catenin needs to associate with several other proteins to perform its functions. Under normal physiological conditions, β-catenin is mainly present in the cell membrane and can participate in a complex with E-cadherin via the armadillo repeats [70–72]. E-cadherin, also known as uvomorulin, is the major cadherin molecule expressed by epithelial cells [73–75]. This complex can mediate the connection of E-cadherin with the cytoskeleton and is involved in cell adhesion. The complex can function to maintain normal cell morphology and inhibit tumor cell invasion and metastasis. E-cadherin can also inhibit β-catenin's transcriptional activities by recruiting β-catenin from its transcriptional complexes [76]. Furthermore, in adherens junctions, β-catenin combines with α-catenin. α-catenin can link adherens junctions with the actin cytoskeleton by associating with filamentous actin directly or indirectly; this cytoskeletal linkage is crucial for the adhesion of cells [52, 77]. In summary, β-catenin provides obvious connections among extracellular signals, cell-cycle management, and gene transcription [23, 78].

### Hot-spot exon mutations of CTNNB1 can drive tumorigenesis

Exon 3 of *CTNNB1* is a key exon encoding serine-threonine phosphorylation sites for GSK-3β that activates degradation of β-catenin [79]. The *CTNNB1* mutations are frequently missense mutations [27]. Nearly all of them have been localized in this hot-spot exon 3, and most of them have occurred at S33, S37, S45, T41, D32, and G34 [6, 57, 80–82]. Of these, S33, S37, and T41 are the phosphorylation sites for GSK-3β; S45 is the phosphorylation site for casein kinase-1; and D32 and G34 are essential for the interaction of β-catenin with Fbw1.

Gene mutations leading to constitutive activation of the Wnt/β-catenin signaling pathway, especially the canonical one, are early events in the development of some cancer cases [83]. Moreover, a high level of β-catenin activity is required for cancer initiation [84]. Initial characterization of mutations of *CTNNB1* and deregulation of the canonical Wnt pathway were in colorectal cancer cases [85, 86]. Since then, these mutations have been described and studied in several other types of malignancies [87–92]. Paul Polakis (2000) has summarized *CTNNB1* mutation spots and rates in human cancers in detail [93]. For example, a mutation of β-catenin (S37F) activates Wnt signaling in several melanoma cell lines [87]. Such mutations have been shown to result in the accumulation of nuclear β-catenin and stabilization of the protein and tumorigenesis [94, 95]. These mutations stabilize β-catenin by abrogating the phosphorylation-dependent interaction of β-catenin with Fbw1 [57]. Fbw1 is a type of F-box protein that is a component of the ubiquitin ligase for β-catenin that associates with β-catenin phosphorylated by casein kinase-1α and GSK-3β resulting in ubiquitination and degradation of β-catenin [57, 96]. In addition, other relatively benign tumors, such as desmoid-type fibromatosis, also have *CTNNB1* mutations and abnormal nuclear β-catenin expression [97, 98].

As described above, under normal physiological conditions, β-catenin is expressed mainly in the cell membrane. In contrast, the expression level of dissociative β-catenin in the cytoplasm is quite low owing to the combination with the destruction complex [7]. Whether the genes upstream from β-catenin are altered, or *CTNNB1* mutates, once a threshold of β-catenin accumulates in the cytoplasm and nucleus, this status reflects abnormal expression of β-catenin [99]. When degradation of β-catenins inhibited the level of dissociated β-catenin in the cytoplasm increases, the protein will then accumulate at high levels in the nucleus. Of all the molecular alterations that lead to disruptions of β-catenin degradation, the most common are mutations that activate β-catenin or inactivate APC [94]. For example, a report suggests *APC* mutations can correlate with high expression levels of β-catenin whereas wild-type *APC* expression can reduce β-catenin levels in colorectal cancer cells [93, 100]. The accumulated β-catenin in the nucleus can then combine with TCF/LEF transcription factors [101–104]. The TCF/β-catenin complex can activate the transcription of proto-oncogenes in humans [105]. The result is activated transcription of downstream cell-proliferation related genes such as *c-myc* and *cyclin D1*. This process promotes cell proliferation [106]. Thus, abnormal expression of β-catenin in the cytoplasm and nucleus is regarded as an important indicator of malignancy [6, 79].

For some cancers, such as endometrioid endometrial carcinoma (EEC), studies have demonstrated that 40-60% of cases exhibited nuclear accumulation of β-catenin in tumor cells [107, 108]. Other studies identified an increase in *CTNNB1* mutations in EEC compared to nonendometrioid endometrial carcinoma cases (NEEC) [27, 109, 110]. Detailed mutational rates are in Table 1. *CTNNB1* mutations seemingly occurr in the early stages of endometrial carcinogenesis [110]. Additionally, authors have described nuclear accumulation of β-catenin and mutations in exon 3 of *CTNNB1* in endometrioid ovarian carcinoma [111–116]. From these aspects, at least in part, both nuclear expression and mutations of β-catenin are characteristic of the endometrioid phenotype [9]. Liu and colleagues (2014) conducted a systematic, comprehensive study of *CTNNB1* mutations in EEC [117]. They performed an integrated analysis including whole-exome sequencing, RNA sequencing, and reverse-phase protein array profiling. The clinical study population consisted of two groups: 271 EEC patients whose data were obtained from The Cancer Genome Atlas and a validation group of 184 EEC patients from The University of Texas MD Anderson Cancer Center. One of the four genes expression clusters in The Cancer Genome Atlas group, cluster II, was enriched with the highest proportion of *CTNNB1* gene mutations. Eighty-seven percent of the tumors in this cluster carried *CTNNB1* mutations. This cluster was associated with young, obese patients with grade 1 or 2 tumors that were early stage at diagnosis (stages I or II) and had decreased expression of estrogen and progesterone receptors. Importantly, this cluster was also associated with decreased overall survival. Table 1 lists the rates of *CTNNB1* mutations in these cases.

**Table 1:** Frequency of *CTNNB1* mutation in different types of EEC and EOC

Histology	Mutation rate (%)	Frequency (N)	Reference
EEC	52	15/29	27
EEC	19	88/454	109
EEC	37	71/192	117
EEC	19	35/183	117
NEEC	0	0/14	27
AEH	14	3/21	110
EOC	50	3/6	111
EOC	54	7/13	112
EOC	16	10/63	114
EOC	31	14/45	114
EOC	26	6/23	115
EOC	38	8/21	116

Although *CTNNB1* mutations have been shown to induce the accumulation of nuclear β-catenin and activate the canonical Wnt pathway, some studies demonstrated that this accumulation can occur without *CTNNB1* mutations [9, 106]. This may indicate that alterations of other molecules or signaling pathways, such as APC, AXIN1, AXIN2, γ-catenin, and the PI3K/AKT signaling pathway, can modulate the Wnt pathway. Mutations in *AXIN1* and *AXIN2* can activate the Wnt pathway and have been associated with the development of a subset of colon, hepatic, and ovarian carcinomas and medulloblastomas [114, 118–120]. Overexpression of γ-catenin has been demonstrated to stabilize and increase the nuclear localization of β-catenin [121]. In addition, activation of PI3K/AKT by different mechanisms, such as GSK-3β inhibition and Ras activation, has been associated with nuclear accumulation of β-catenin in some cancers [122–124]. In some cancer cell lines, PI3K/AKT signaling activated β-catenin–mediated transcription [125]. Inhibition of PI3K/AKT signaling reduced Wnt signaling in medulloblastoma cells [126]. Similarly, in colon cancers, hepatocyte growth factor and MAPK signaling pathway may be regulators of Wnt signaling to mediate tumor progression [124, 127–130].

There are also critical upstream regulators of β-catenin, specifically p53 and microRNAs (miRNAs), that impact canonical Wnt signaling. As a tumor suppressor, loss of p53 function can activate canonical Wnt pathway by its transcriptional activity [131]. miRNAs are small and non-coding RNAs that can interact with untranslated regions of mRNA targets; repressing gene expression post-transcriptionally [132, 133]. Studies in recent years have found that several miRNAs could regulate the canonical Wnt pathway. For example, miRNA-34 suppressed the transcriptional activity of β-catenin/TCF complexes by targeting the untranslated regions of Wnt pathway-regulated genes [131]. Additionally, miRNA-370-3p could inhibit downstream genes of Wnt pathway, like *cyclin D1* and *c-myc*, by binding with the 3’-untranslated region of β-catenin mRNA directly [134]. Roman Anton and colleagues (2011) performed a systematic screen of 470 miRNAs and identified 38 miRNAs that either activate, or repress the canonical Wnt pathway [135]. miRNA-1 and miRNA-613 acted upstream of β-catenin while miRNA-25 functioned at the level of β-catenin. From these studies, we can conclude that other factors in addition to *CTNNB1* mutation can activate the canonical Wnt pathway.

## THE CONNECTION BETWEEN β-CATENIN EXPRESSION AND IMMUNOSUPPRESSION IN THE CANCER MICROENVIRONMENT

**Figure 3:**
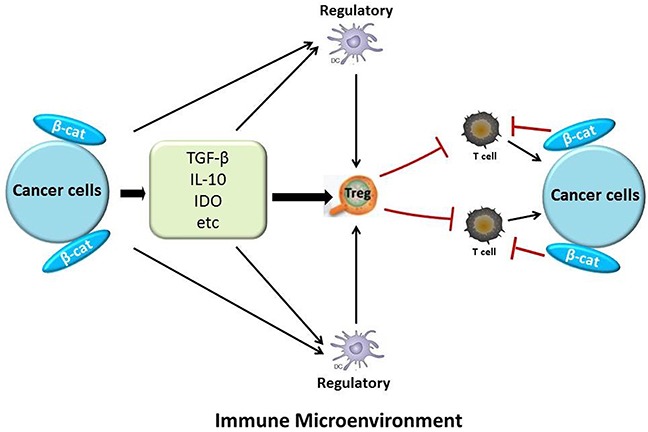
The relationship between β-catenin and immunosuppression in the tumor microenvironment Cancer cells can induce relevant immunosuppressive molecules and cells, such as TGF-β, IL-10, indoleamine 2,3-dioxygenase (IDO), Tregs, and regulatory DCs. They can inhibit T cells, which have toxic effects on cancer cells. Also, cancer-intrinsic β-catenin signaling can decrease T-cell infiltration. β-catenin also helps cancer cells secrete immunosuppressive molecules.

Many types of tumors, including hematological malignancies and melanoma have been treated with immunotherapy [136, 137]. However, this approach often has insufficient antitumor effects [138, 139]. One important obstacle of this treatment approach is immunoescape through localized immunosuppression and immunoresistance, which is one of the major malignant characteristics of cancer cells [140]. Cancer cells can activate some immunosuppressive cells such as regulatory dendritic cells (DCs), and regulatory T cells (Tregs) through production of many immunosuppressive molecules such as transforming growth factor (TGF)-β, interleukin (IL)-10 [140]. Researchers have found that β-catenin correlated to the infiltration of immune cells in the tumor microenvironment (Figure 3) [141, 142]. Tumor-intrinsic β-catenin signaling could inhibit T-cell infiltration in melanoma models and in patient-derived biopsies [141]. Melanoma gene microarray data have suggested that an activated Wnt/β-catenin signal in the cancer microenvironment can be correlated with a lack of an immune cell infiltration [143]. Additionally, overexpression and mutations of β-catenin can cause the production of IL-10 at high levels in melanoma cells [138]. Investigators have observed similar events in EC. In the immune microenvironment of EC, cancer cells can secrete many immunosuppressive molecules, such as IL-10, TGF-β, and indoleamine 2,3-dioxygenase [140, 144] that could suppress the differentiation, maturation, and function of DCs and effector T cells [145]. Other immunosuppressive cells, such as regulatory DCs and Tregs, can be induced not only by TGF-β, IL-10 and other immunosuppressive molecules, but also by EC directly [140, 146]. Regulatory DCs also can induce Tregs. In addition, Tregs can potently suppress T-cell-mediated immune responses [147–150]. According to a statistical analysis, *CTNNB1* mutations were associated with TGF-β2, which contributes to tumor progression. These mutations also influenced the numbers of cytotoxic cells that can kill tumor cells, such as lymphocytes and macrophages [117]. From these data we may conclude that β-catenin has some connections with immunosuppresion in the cancer environment.

## THE CONNECTION BETWEEN β-CATENIN EXPRESSION AND METABOLIC REGULATION

Metabolic regulation is essential to tumorigenesis, and metabolic reprogramming is one of the hallmarks of tumor cells [151]. Metabolic reprogramming in cancer cells may contribute in targeting therapy resistance [152]. Via the Warburg effect, glucose metabolism can confer a powerful growth advantage to tumor cells from oxidative phosphorylation to glycolysis regardless of the oxygen supply [153–155]. Tumor cells need more glucose than normal cells to meet their elevated anabolic and energy demands, and are more sensitive to glucose deprivation, a phenomenon known as glucose addiction, [155, 156]. Tumor cells seem to prefer the glycolytic pathway to oxidative phosphorylation to produce ATP to support their anabolic production of biomass [157–159]. This preference may be a protective strategy against reactive oxygen species. Hence, glycolysis can protect genome integrity during DNA replication [160–162].

**Figure 4:**
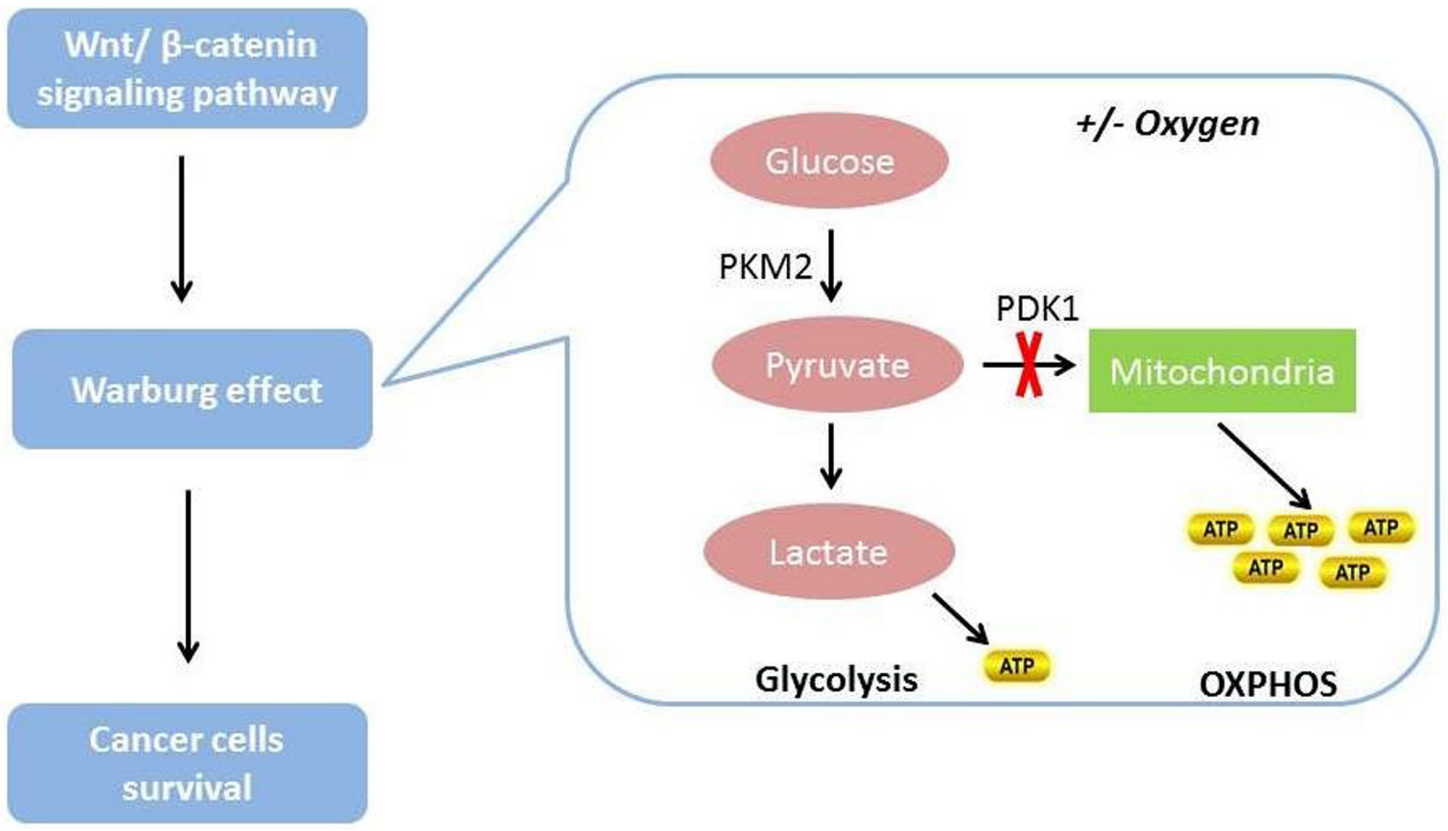
The relationship between Wnt/β-catenin signaling pathway and glucose metabolism in cancer cells The Wnt/β-catenin signaling pathway can induce the Warburg effect and then promote cancer cell survival. Cancer cells prefer to use the Warburg effect to satisfy their energy demands regardless of the presence or absence of oxygen.

The Wnt/β-catenin signaling pathway can induce the Warburg effect and establish metabolic zonation (Figure 4) [153, 163–165]. However, once this signaling pathway is blocked, the free: bound NADH ratio and lactate production decrease. Decreases in both can indicate decreased glycolysis. In addition, the expression of two downstream target genes, the *lactate transporter MCT-1 (SLC16A1)* and *pyruvate dehydrogenase kinase, isozyme 1*, are also downregulated due to this blockage [166]. Pyruvate dehydrogenase kinase, isozyme 1 can phosphorylate and inhibit the pyruvate dehydrogenase complex in mitochondria, inhibiting the conversion of pyruvate to acetyl-CoA. This means that the next step, oxidative phosphorylation is blocked [167]. Therefore, pyruvate dehydrogenase kinase, isozyme 1 plays an important role in promotion of the glycolytic phenotype. Pyruvate kinase M2, one of the key enzymes in glucose metabolism, is highly expressed in human cancer cells and can stimulate glycolysis [168]. Authors have reported that nuclear pyruvate kinase M2 can activate β-catenin transactivation upon epidermal growth factor receptor activation [168].

Metformin has been used to treat hyperglycemia and type 2 diabetes mellitus [169]. This drug mainly increases insulin sensitivity, suppresses hepatic glucose production, reduces glucose absorption from the intestines, and reduces the incidence of lipolysis in adipocytes [170, 171]. Metformin also has antineoplastic efficacy [172]. It can activate adenosine monophosphate-activated protein kinase (APMK), which maintains cellular energy homeostasis in response to various stimuli [173, 174]. In addition, sustained AMPK activation has been linked with apoptosis [175]. AMPK inhibits the expression of *bone morphogenic protein and the activin membrane-bound inhibitor (Bambi)*, which can induce Wnt/β-catenin signaling pathway leading to cell proliferation and survival [173, 176]. Also, a positive feedback loop exists between Bambi and this pathway [173]. Taken together, these findings indicate that the Wnt/β-catenin signaling pathway has a relationship with the metabolicnetwork. Metabolic control can regulate the progression of tumors, which is accomplished by regulating the Wnt/β-catenin signaling pathway.

## TARGETED APPLICATIONS OF β-CATENIN FOR CANCER TREATMENT

Researchers revealed that β-catenin was required for the development of triple-negative breast cancer that is estrogen-dependent [177]. In their study, they found that cell migration, colony formation, and epithelial-to-mesenchymal transition *in vitro* and tumorigenesis *in vivo* were regulated by β-catenin in triple-negative breast cancer. In addition, they proved that the transcriptional activity of β-catenin was quite important for the chemosensitivity of triple-negative breast cancer cells. So at least in part, β-catenin can be considered as one of therapeutic targets for this cancer.

Past studies have elaborated that antagonists of β-catenin or other proteins in the Wnt pathway could block this pathway and inhibit cell proliferation in many different tumor types [47, 95, 178–185]. Table 2 lists these antagonists and their possible mechanisms of action. For example, PKF115-584 is a small-molecule antagonist of the TCF/β-catenin complex [183]. It can decrease both cytoplasmic and nuclear β-catenin expression levels [186–188]. This antagonist downregulated the mRNA and protein expression levels for the critical proliferative genes of pancreatic neuroendocrine tumor cells [95]. Fiskus and colleagues (2015) used BC2059 to treat acute myeloid leukemia cells and found induction of apoptosis of the leukemia cells [47]. BC2059 is a small molecule that is considered to be an anthraquinone oxime analog. It can disrupt the binding of β-catenin to transducin β-like 1 and promote β-catenin degradation.

**Table 2:** The antagonists of β-catenin or other proteins in Wnt pathway and their possible mechanisms

Name	Possible mechanisms
PKF115-584^183^	Antagonist of the TCF/β-catenin complex Decreases both cytoplasmic and nuclear β-catenin expression
BC2059^47^	Anthraquinone oxime analog Disrupts the binding of β-catenin to transducin β-like 1; Promotes β-catenin degradation
LF3^180^	Inhibits the β-catenin/TCF4 interaction Suppresses cell motility, cell-cycle progression, and the overexpression of Wnt target genes; Blocks the self-renewal capacity of cancer stem cells
ICG-001^181^	Inhibits β-catenin/CREBBP interaction Downregulates Wnt target genes
Salinomycin^185^	Small molecule inhibitor of LRP6 Downregulates Wnt target genes and cause cancer cell death
Calphostin C^182^	Antagonist of the TCF/β-catenin complex Degrades β-catenin via a proteasome-dependent
Xanthothricin^182^	Antagonist of the TCF/β-catenin complex Degrades β-catenin via a proteasome-dependent
FH535^184^	Suppresses β-catenin /TCF-mediated transcription

Besides the specific antagonists, hormones can influence the expression of β-catenin. For example, progesterone can counteract the accumulation of β-catenin in the nucleus and inhibit the Wnt/β-catenin signaling pathway [44, 108]. This hormone can also induce the expression of two inhibitors of the Wnt/β-catenin signaling pathway, DKK1 and FOXO1 [189–191]. DKK1 can inhibit Wnt/β-catenin signaling by binding to the Wnt co-receptors LRP5 and LRP6 [192]. For FOXO1, investigators found that it could inhibit this signaling by binding to β-catenin [191–193].

## CONCLUSION

In summary, the important canonical Wnt/β-catenin signaling pathway exerts potent biological functions. As evidence, induction of tumorigenesis occurs when any step in this pathway is deregulated. β-catenin is the key intracellular transducer of this signaling pathway. Abnormal expression and mutations of β-catenin have been regarded as important signs of malignancy [6, 79]. Researchers have described and studied its mutations in many cancers, including colon cancer, melanoma, cervical carcinoma, endometrial cancer (EC), hepatoblastoma, and primitive neuroectodermal brain tumors. β-catenin mutations also can be considered to be the initiating factors for tumorigenesis and are positively related to poor survival, although the specific mechanism of this tumorigenesis deserves further study [8, 9, 112, 194]. Hence, we conclude that if we specifically inhibit the abnormal expression of β-catenin, cancers could be treated more effectively.

Another noteworthy problem about β-catenin is that some studies demonstrated that β-catenin activation alone could not induce spontaneous development of hepatocellular carcinoma in mouse models [195]. β-catenin`s role of tumor promotion requires the participation of other proteins, such as constitutive androstane receptor, which is a primary regulator of drug metabolism and detoxification. As a multifunctional protein, β-catenin can participate in a variety of physiological activities. As we described above, β-catenin can enhance the survival of Tregs and induce the secretion of immunosuppressive cytokines such as IL-10 and TGF-β. Treatments focusing on immunosuppression in the cancer microenvironment resulting from β-catenin should deserve more attention. The Warburg effect is well known to be one of the hallmarks of tumor cells. These cells prefer to use glycolysis rather than oxidative phosphorylation for producing ATP regardless of the sufficiency of oxygen. Once the Wnt/β-catenin signaling pathway is blocked, the production of some downstream metabolites of glycolysis, such as lactate, will decrease. Given this, we can conclude that the progression of tumors can be limited by regulating their glucose metabolism. β-catenin, therefore, seems to be an ideal therapeutic target for cancer. Besides the antagonists of β-catenin, hormones such as progesterone can also be of worth for cancer treatments. Certainly, more effective antagonists of β-catenin should be developed and applied in the clinic for cancer treatment. In the near future, early carcinogenesis may be initially inhibited by controlling abnormal β-catenin activity.

## Abbreviations

APC: adenomatosis polyposis coli
CK-1: casein kinase-1α
PP2A: protein phosphatase 2A
GSK-3β: glycogen synthase kinase-3β
TCF/LEF: T-cell factor/lymphoid enhancer factor
CBP: CREB-binding protein
LRP: low-density lipoprotein receptor-related protein
PI3K: phosphoinositide 3-kinase
MAPK: mitogen-activated protein kinase
EEC: endometrioid endometrial carcinoma
NEEC: nonendometrioid endometrial carcinoma
miRNAs: microRNAs
DCs: dendritic cells
Tregs: regulatory T cells
TGF: transforming growth factor
IL: interleukin
APMK: adenosine monophosphate-activated protein kinase
Bambi: bone morphogenic protein and the activin membrane-bound inhibitor
EC: endometrial cancer
AEH: atypical endometrial hyperplasia
EOC: endometrioid ovarian cancer
